# In utero exposure to breast cancer treatment: a population-based perinatal outcome study

**DOI:** 10.1038/s41416-019-0563-x

**Published:** 2019-09-06

**Authors:** Nadom Safi, Antoinette Anazodo, Jan E. Dickinson, Kei Lui, Alex Y. Wang, Zhuoyang Li, Elizabeth A. Sullivan

**Affiliations:** 1 Faculty of Health, University of Technology Sydney, Sydney, NSW Australia; 2 The Kids Cancer Centre, Sydney Children’s Hospital, Sydney, NSW Australia; 3 Nelune Comprehensive Cancer Centre, Prince of Wales Hospital, Sydney, NSW Australia; 4 Faculty of Health and Medical Sciences, Division of Obstetrics and Gynaecology, The University of Western Australia, Perth, WA Australia; 5 Department of Newborn Care, Royal Hospital for Women, Sydney, Australia; 6 School of Women’s and Child’s Health, University of New South Wales, Sydney, Australia; 7 Faculty of Health and Medicine, University of Newcastle, Callaghan, NSW Australia

**Keywords:** Epidemiology, Outcomes research, Cancer epidemiology

## Abstract

Chemotherapy during a viable pregnancy may be associated with adverse perinatal outcomes. We conducted a prospective cohort study to examine the perinatal outcomes of babies born following in utero exposure to chemotherapy in Australia and New Zealand. Over 18 months we identified 24 births, of >400 g and/or >20-weeks’ gestation, to women diagnosed with breast cancer in the first or second trimesters. Eighteen babies were exposed in utero to chemotherapy. Chemotherapy commenced at a median of 20 weeks gestation, for a mean duration of 10 weeks. Twelve exposed infants were born preterm with 11 by induced labour or pre-labour caesarean section. There were no perinatal deaths or congenital malformations. Our findings show that breast cancer diagnosed during mid-pregnancy is often treated with chemotherapy. Other than induced preterm births, there were no serious adverse perinatal outcomes.

## Background

The management of cancer diagnosed during pregnancy poses unique challenges in optimising maternal and infant outcomes. One of these challenges is choosing the optimal treatment regimen to balance the benefit to the women and the potential risks of adverse outcomes for the foetus.^1^ Timing of treatment initiation is also challenging, especially if cancer diagnosis is early in the first trimester as foetal exposure to chemotherapy during the period of organogenesis has been associated with an increased risk of congenital malformations.^2,3^

This study describes the perinatal outcomes of babies of women diagnosed with breast cancer during the first or second trimesters of pregnancy by whether exposed to in utero systemic chemotherapy or not.

## Methods

A population-based prospective cohort study design was conducted in Australia and New Zealand using the Australasian Maternity Outcomes Surveillance System (AMOSS).^4^ We identified babies born to women with a confirmed diagnosis of breast cancer during pregnancy through monthly surveillance between January 2013 and June 2014. Eligible births included live or stillborn babies of at least 400 g or 20 weeks gestation whether exposed to chemotherapy or not. Data were collected on maternal and cancer care, and perinatal outcomes.

Perinatal outcomes included: stillbirth, neonatal death, major congenital malformations, preterm birth (<37 completed weeks of gestation), low birthweight (<2,500 grams) and small for gestational age (birthweight <10th percentile for gestational age).^5^

Chi-square, Fisher’s exact test, Fisher-Freeman-Halton test, and independent sample *t*-test were used to investigate the difference in outcomes of babies stratified by in utero exposure to chemotherapy.

## Results

Of the 24 babies born to women diagnosed with breast cancer during the first and second trimesters of pregnancy, 18 (75%) were exposed to chemotherapy, and six were not (detailed in Supplementary Table 1). Demographic and treatment characteristics of the 24 women are shown in Supplementary Tables 1 and 2.

The types of systemic chemotherapeutic agents used during the pregnancies are listed in Supplementary Table 3. The median gestational age at first in utero exposure was 20 weeks (range 13–31). Fourteen (77.8%) of the 18 babies had their first exposure in the second trimester and four (22.2%) in the third trimester. All 18 babies were exposed to a minimum of two therapeutic agents with a mean duration of exposure of 10.4 ± 5.8 weeks. All babies were exposed to alkylating agents; either nitrogen mustard (Cyclophosphamide) or platinum compounds (Carboplatin), 16 (88.9%) were exposed to anthracyclines (Doxorubicin or Epirubicin), 10 (55.6%) to taxanes (Paclitaxel or Docetaxel) and 1 (5.6%) to Fluorouracil.

The mean gestational age at birth for the 18 chemotherapy exposed babies was 35.7 ± 2 weeks, significantly lower than that for the six non-exposed babies (mean 38.8 ± 1.5 weeks) (*P* = 0.002) (Table 1). There were no stillbirths, diagnosed congenital malformations or neonatal deaths in any of the 24 babies. The need for resuscitation was seen in the two babies exposed to Tamoxifen combined systemic therapy (Cyclophosphamide, Doxorubicin and Docetaxel and Tamoxifen; and Paclitaxel, Carboplatin and Tamoxifen). The former was female born following induction at 36 weeks, birthweight 2480 g Apgar score at 5 min of 8 and resuscitated with a continuous positive airway pressure (CPAP) mask, however, discharged home without the need for admission to neonatal intensive care (NICU) or Special Care Nursery (SCN). The latter was a boy delivered at 34 weeks by CS (birthweight 2240 g, Apgar score at 5 min of 8) and required resuscitation with a continuous positive airway pressure (CPAP) mask and admission to the SCN.

**Table 1:** Perinatal outcomes amongst the 24 babies

	Exposed	Non-exposed	*P* value
	(*n* = 18)	(*n* = 6)	
Live births	18 (100)	6 (100)	NA
Neonatal deaths^a^	0 (0)	0 (0)	NA
Preterm (<37 weeks)			
Yes	12 (66.7)	0 (0)	0.014
*<32 weeks*	*1* (*5.6)*	*0* (*0)*	
*33–* < *37 weeks*	*11* (*61.1)*	*0* (*0)*	
No	6 (33.3)	6 (100)	
Small for gestational age	2 (11.1)	0 (0)	1.000
Low birthweight (<2500 g)	9 (50)	0 (0)	0.052
Resuscitation			
Yes	6 (33.3)	0 (0)	0.277
*Neopuff or CPAP mask only*	*3* (*16.7)*	*0* (*0)*	
*Oxygen*	*1* (*5.6)*	*0* (*0)*	
*Neopuff or CPAP mask* *+* *Suction + Oxygen*	*2* (*11.1)*	*0* (*0)*	
No	12 (66.7)	6 (100)	
Respiratory support			
Yes^b^	1 (5.6)	0 (0)	1.000
No	16 (88.9)	6 (100)	
Not known	1 (5.6)	0 (0)	
Apgar score (5 min)			
8	5 (27.8)	0 (0)	0.348
9	10 (55.6)	5 (83.3)	
10	3 (16.7)	1 (16.7)	
Admission to NICU/SCN	9 (50)	1 (16.7)	0.341
Breastfeeding initiated			
Yes	6 (33.3)	5 (83.3)	0.061
No	12 (66.7)	1 (16.7)	

^a^During hospital stay only

^b^CPAP mask only

Neopuff Infant T-Piece Resuscitator

*CPAP* Continuous Positive Airways Pressure

A third baby exposed to Trastuzumab, Docetaxel and Cyclophosphamide was born vaginally following induction at 36 weeks (birthweight 2380 g; Apgar score of 10) and was admitted to SCN with mild respiratory distress before being discharged home on day 4.

Ten of the babies were exposed to Taxanes in addition to other chemotherapeutic agents. However, their perinatal outcomes did not significantly differ from those who had exposed to non-Taxane chemotherapy (Supplementary Table 4).

## Discussion

In this analysis, we examined the effect of in utero exposure to chemotherapy on perinatal outcomes. As expected, the gestation at diagnosis influenced the decision on the timing of chemotherapy and the non-use of radiotherapy during pregnancy. All cases in our study whether exposed to chemotherapy or not were diagnosed in the first or second trimesters. The other factors influencing management decisions are the grading and staging of breast cancer. Of note, none of the non-exposed babies’ mothers had distant metastasis and none had a preterm birth.

It is recognised that management decisions are often a delicate balance in considering the treatment impacts on both the maternal and foetal health during the pregnancy. In this study, apart from preterm birth, there were no serious adverse perinatal outcomes in the 18 babies exposed to chemotherapy nor in the six non-exposed babies. There was no perinatal death or congenital malformations.

The majority of exposed babies were exposed to cyclophosphamide and doxorubicin, with one baby exposed to trastuzumab and two others to tamoxifen. This is consistent with the other studies in which the babies were mainly exposed to a combination of cyclophosphamide and doxorubicin.^6–9^

Tamoxifen is contraindicated during pregnancy.^10^ The two babies who were exposed to tamoxifen in our study were born without congenital malformations. However, due to the small number of babies exposed to tamoxifen in our study, we were unable to recommend the use of tamoxifen during pregnancy.

Trastuzumab is contraindicated during pregnancy, as it has been associated with oligohydramnios and renal impairment in the foetus.^11^ We were unable to confirm this association as in our study only one baby was exposed to trastuzumab in the third trimester.

In agreement with other studies,^2^ our results show a significantly higher rate of preterm births among babies exposed to systemic therapy during pregnancy compared to the non-exposed babies (12 out of 18 vs 0 out of 6).

Morbidities in neonates (low birthweight and admission to NICU/SCN) in our study were directly linked to preterm birth. Similar to the previous studies,^2,9^ the leading cause of preterm birth amongst the exposed group in our study is iatrogenic to facilitate maternal systemic chemotherapy postpartum (supplementary fig. 1).

There is a growing evidence on the safety of exposure to anthracyclines containing regimens after the first trimester; however, it is limited for the other chemotherapeutic agents and the non-chemotherapy systemic treatment. There is a need for standardised information on the maternal-foetal exposure and outcomes of chemotherapy and other systemic anticancer agents use in pregnancy that is collated internationally into a database for use in informing clinical practice and research worldwide.

A major strength of this cohort study is its population-design of all cases in Australia and New Zealand during the study period. Limitations include the rarity of the condition, the low uptake of chemotherapy during pregnancy and the follow-up period being restricted to the perinatal period.

## Conclusion

Our study provides assurance that there were no congenital abnormalities or perinatal deaths among the 18 babies exposed to at least two different chemotherapy agents during pregnancy. The directionality of our findings is consistent with the two largest studies in the international literature, particularly regarding preterm birth.^6,7^ Larger observational studies are needed to provide better information on in utero exposure and outcomes following chemotherapy to inform gestational breast cancer management.

## Supplementary information

Supplementary materials

## Data Availability

Data will be available from the corresponding author on reasonable request.

## References

[1] Morice P, Uzan C, Uzan S (2012). Cancer in pregnancy: a challenging conflict of interest. Lancet.

[2] Amant F, Van Calsteren K, Halaska MJ, Gziri MM, Hui W, Lagae L (2012). Long-term cognitive and cardiac outcomes after prenatal exposure to chemotherapy in children aged 18 months or older: an observational study. Lancet Oncol..

[3] Albright CM, Wenstrom KD (2016). Malignancies in pregnancy. Best Pract. Res. Clin. Obstet. Gynaecol..

[4] AMOSS. Australasian Maternity Outcomes Surveillance System Annual Report 2010–2011.https://npesu.unsw.edu.au/surveillance/australasian-maternityoutcomes-surveillance-system-annual-report-2010%E2%80%932011. Accessed 22 August, 2019.

[5] Australian Institute of Health and Welfare. *Australia’s mothers and babies 2015—in brief*. *Perinatal statistics series no. 33. Cat no. PER 91*. (AIHW, Canberra, 2017)

[6] Amant F, Vandenbroucke T, Verheecke M, Fumagalli M, Halaska MJ, Boere I (2015). Pediatric outcome after maternal cancer diagnosed during pregnancy. N Engl J Med.

[7] Cardonick EH, Gringlas MB, Hunter K, Greenspan J (2015). Development of children born to mothers with cancer during pregnancy: comparing in utero chemotherapy-exposed children with nonexposed controls. Am J Obstet Gynecol.

[8] Hahn KM, Johnson PH, Gordon N, Kuerer H, Middleton L, Ramirez M (2006). Treatment of pregnant breast cancer patients and outcomes of children exposed to chemotherapy in utero. Cancer.

[9] Loibl S, Han SN, von Minckwitz G, Bontenbal M, Ring A, Giermek J (2012). Treatment of breast cancer during pregnancy: an observational study. Lancet Oncol.

[10] Peccatori FA, Lambertini M, Scarfone G, Del Pup L, Codacci-Pisanelli G (2018). Biology, staging, and treatment of breast cancer during pregnancy: reassessing the evidences. Cancer Biol Med.

[11] Shachar SS, Gallagher K, McGuire K, Zagar TM, Faso A, Muss HB (2017). Multidisciplinary management of breast cancer during pregnancy. Oncologist.

