# Supplementary material for: In utero exposure to breast cancer treatment: a population-based perinatal outcome study
Source: Br J Cancer. 2019 Sep 6;121(8):719–21. doi: 10.1038/s41416-019-0563-x (PMC6888827; doi:10.1038/s41416-019-0563-x)
Supplement: Supplementary file 1 — Supplementary materials [file 41416_2019_563_MOESM1_ESM.docx]

**Supplementary Table 1: Maternal demographics**

|  | Exposed | Non-exposed | P value |
| --- | --- | --- | --- |
|  | (n=18) | (n=6) |  |
| Country |  |  |  |
| Australia | 15(83.3) | 5(83.3) | 1.000 |
| New Zealand | 3(16.7) | 1(16.7) |  |
| Age (years) |  |  |  |
| <35 | 9(50) | 3(50) | 1.000 |
| ≥35 | 9(50) | 3(50) |  |
| BMI (kg/m^2^) |  |  |  |
| 18.50 - 24.99 | 12(66.7) | 4(66.7) | 1.000 |
| ≥25.00 | 5(27.8) | 2(33.3) |  |
| Unknown | 1(5.6) | 0(0) |  |
| Hospital Sector |  |  |  |
| Public | 11(61.1) | 5(83.3) | 0.621 |
| Private | 7(38.9) | 1(16.7) |  |
| Parity |  |  |  |
| 0 | 7(38.9) | 4(66.7) | 0.357 |
| >1 | 11(61.1) | 2(33.3) |  |
| Smoking status |  |  |  |
| Never smoked | 9(50) | 5(83.3) | 0.319 |
| Quit smoking before becoming pregnant | 4(22.2) | 0(0) |  |
| Smoking during pregnancy | 1(5.6) | 1(16.7) |  |
| Not known | 4(22.2) | 0(0) |  |
| ART* |  |  |  |
| Yes | 0(0) | 0(0) | N/A |
| No | 18(100) | 5(83.3) |  |
| Not known | 0(0) | 1(16.7) |  |

*ART = assisted reproductive technology

**Supplementary Table 2: Maternal cancer characteristics, tumour treatment and obstetric management**

|  | Exposed | Non-exposed | P value |
| --- | --- | --- | --- |
|  | (n=18) | (n=6) |  |
| Tumour grade |  |  |  |
| Low | 0(0) | 3(50) | 0.030 |
| Intermediate | 3(16.7) | 0(0) |  |
| High | 12(66.7) | 3(50) |  |
| Not known | 3(16.7) | 0(0) |  |
| Lymphovascular Involvement |  |  |  |
| Yes | 7(38.9) | 0(0) | 0.123 |
| No | 9(50) | 5(83.3) |  |
| Not known | 2(11.1) | 1(16.7) |  |
| Estrogen receptor status |  |  |  |
| Positive | 12(66.7) | 4(66.7) | 1.000 |
| Negative | 5(27.8) | 1(16.7) |  |
| Not known | 1(5.6) | 1(16.7) |  |
| Progestrone receptor status |  |  |  |
| Positive | 9(50) | 4(66.7) |  |
| Negative | 8(44.4) | 1(16.7) | 0.360 |
| Not known | 1(5.6) | 1(16.7) |  |
| HER 2 status |  |  |  |
| Positive | 4(22.2) | 0(0) | 0.546 |
| Negative | 13(72.2) | 4(66.7) |  |
| Not known | 1(5.6) | 2(33.3) |  |
| Metastatic Disease |  |  |  |
| Yes | 6(33.3) | 0(0) | 0.144 |
| No | 11(61.1) | 6(100) |  |
| Not known | 1(5.6) | 0(0) |  |
| Surgery During Pregnancy |  |  |  |
| Yes | 15(83.3) | 5(83.3) | 1.000 |
| No, delayed until end of pregnancy | 3(16.7) | 1(16.7) |  |
| Radiotherapy During Pregnancy |  |  |  |
| No, not recommended | 6(33.3) | 3(50) | 0.635 |
| No, delayed until end of pregnancy | 12(66.7) | 3(50) |  |
| Postpartum Systemic Therapy |  |  |  |
| Yes | 17(94.4) | 2(33.3) | 0.021 |
| No | 1(5.6) | 3(50) |  |
| Not known | 0(0) | 1(16.7) |  |
| Corticosteroid for fetal lung maturity |  |  |  |
| Yes | 10(55.6) | 0(0) | 0.015 |
| No | 6(33.3) | 6(100) |  |
| Not known | 2(11.1) | 0(0) |  |
| Induction of labour |  |  |  |
| Yes | 10(55.6) | 5(83.3) | 0.531 |
| No/not applicable | 8(44.4) | 1(16.7) |  |
| Method of birth |  |  |  |
| Vaginal birth | 11(61.1) | 4(66.6) | 1.000 |
| Caesarean section | 7(38.9) | 2(33.3) |  |

**Supplementary Table 3: Systemic therapeutic agents during pregnancy.**

|  | Timing of therapy | |  |
| --- | --- | --- | --- |
|  | 2nd Trimester  (13-27 weeks)  (n=14) | 3rd Trimester  (28-40) weeks  (n=4) | Total  (n=18) |
|  | n* (%) | n* (%) | n* (%) |
| Cyclophosphamide |  |  |  |
| Yes | 13(92.9) | 4(100) | 17(94.4) |
| No | 1(7.1) | 0(0) | 1(5.6) |
| Carboplatin |  |  |  |
| Yes | 1(7.1) | 0(0) | 1(5.6) |
| No | 13(92.9) | 4(100) | 17(94.4) |
| Docetaxel |  |  |  |
| Yes | 2(14.3) | 1(25) | 3(16.7) |
| No | 12(85.7) | 2(50) | 14(77.8) |
| Not stated | 0(0) | 1(25) | 1(5.6) |
| Doxorubicin |  |  |  |
| Yes | 12(85.7) | 3(75) | 15(83.3) |
| No | 2(14.3) | 0(0) | 2(11.1) |
| Not stated | 0(0) | 1(25) | 1(5.6) |
| Epirubicin |  |  |  |
| Yes | 1(7.1) | 0(0) | 1(5.6) |
| No | 13(92.9) | 2(50) | 15(83.3) |
| Not stated | 0(0) | 2(50) | 2(11.1) |
| Fluorouracil |  |  |  |
| Yes | 1(7.1) | 0(0) | 1(5.6) |
| No | 13(92.9) | 2(50) | 15(83.3) |
| Not stated | 0(0) | 2(50) | 2(11.1) |
| Paclitaxel |  |  |  |
| Yes | 6(42.9) | 1(25) | 7(38.9) |
| No | 8(57.1) | 2(50) | 10(55.6) |
| Not stated | 0(0) | 1(25) | 1(5.6) |
| Tamoxifen |  |  |  |
| Yes | 2(14.3) | 0(0) | 2(11.1) |
| No | 12(85.7) | 2(50) | 14(77.8) |
| Not stated | 0(0) | 2(50) | 2(11.1) |
| Trastuzumab |  |  |  |
| Yes | 0(0) | 1(25) | 1(5.6) |
| No | 14(100) | 2(50) | 16(88.9) |
| Not stated | 0(0) | 1(25) | 1(5.6) |

*Babies may have been exposed to more than one therapeutic agent.

**Supplementary Table 4:** **Perinatal outcomes amongst the 18 babies exposed to chemotherapy based of their exposure to Taxanes.**

|  | Taxanes yes | Taxanes no | P value |
| --- | --- | --- | --- |
|  | (n=10) | (n=8) |  |
| Live births | 10(100) | 8(100) | NA |
| Neonatal deaths* | 0(0) | 0(0) | NA |
|  |  |  |  |
| Preterm (<37 weeks) |  |  |  |
| Yes | 7(70) | 5(62.5) | 1.000 |
| *<32 weeks* | *0(0)* | *1(12.5)* |  |
| *33-<37 weeks* | *7(70)* | *4(50)* |  |
| No | 3(30) | 3(37.5) |  |
| Small for gestational age | 1(10) | 1(12.5) | 1.000 |
| Low birthweight (<2500 g) | (0) | (0) | 1.000 |
| Resuscitation |  |  |  |
| Yes | 4(40) | 2(25) | 1.000 |
| *Neopuff or CPAP mask only* | *2(20)* | *1(12.5)* |  |
| *Oxygen* | *1(10)* | *0(0)* |  |
| *Neopuff or CPAP mask + Suction + Oxygen* | *1(10)* | *1(12.5)* |  |
| No | 6(60) | 6(75) |  |
| Respiratory support |  |  |  |
| Yes** | 1(10) | 0(0) | 1.000 |
| No | 9(90) | 7(87.5) |  |
| Not known | 0(0) | 1(12.5) |  |
| Apgar score (5 minutes) |  |  |  |
| 8 | 3(30) | 2(25) | 0.241 |
| 9 | 4(40) | 6(75) |  |
| 10 | 3(30) | 0(0) |  |
| Admission to NICU/SCN | 5(50) | 4(50) | 1.000 |
| Breastfeeding initiated |  |  |  |
| Yes | 3(30) | 3(37.5) | 1.000 |
| No | 7(70) | 5(62.5) |  |

**Supplementary Figure 1** Mode of birth and postpartum maternal treatment for preterm babies exposed to systemic therapy.


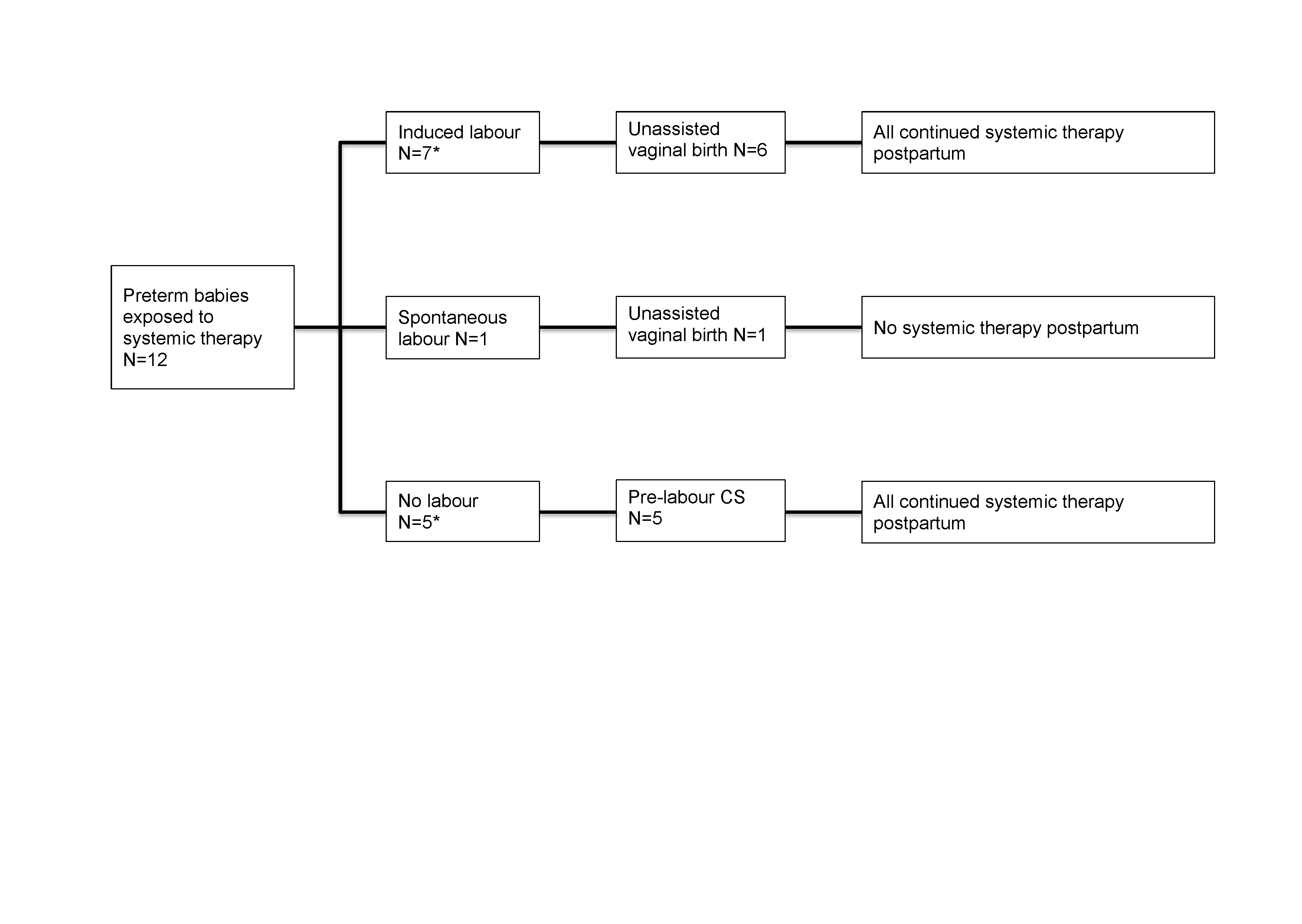


*One case with failure of induction also included in no labour category.
